# Ethnic differences in childhood right and left cardiac structure and function assessed by cardiac magnetic resonance imaging

**DOI:** 10.1007/s00431-020-03869-0

**Published:** 2020-11-10

**Authors:** Wouter J. van Genuchten, Liza Toemen, Arno A. W. Roest, Meike W. Vernooij, Romy Gaillard, Willem A. Helbing, Vincent W. V. Jaddoe

**Affiliations:** 1 The Generation R Study Group (Na 2915), Erasmus MC, University Medical Center, PO Box 2040, 3000 CA Rotterdam, the Netherlands; 2 Department of Pediatrics, Erasmus MC, University Medical Center, Rotterdam, the Netherlands; 3 Department of Pediatrics, Leiden University Medical Center, Leiden, the Netherlands; 4 Department of Radiology & Nuclear Medicine, Erasmus MC, University Medical Center, Rotterdam, the Netherlands

**Keywords:** Pediatrics, Cardiovascular risk, Cardiovascular imaging, MRI, Ethnic groups

## Abstract

Ethnic differences in cardiovascular risk factors and disease are well-known and may originate in early-life. We examined the ethnic differences in cardiac structure and function in children using cardiac magnetic resonance imaging in a European migrant population, and whether any difference was explained by early life factors. We used a prospective population-based cohort study among 2317 children in Rotterdam, the Netherlands. We compared children from Dutch (73%), Cape Verdean (3.5%), Dutch Antillean (3.3%), Moroccan (6.1%), Surinamese-Creoles (3.9%), Surinamese-Hindustani (3.4%), and Turkish (6.4%) background. Main outcomes were cMRI-measured cardiac structures and function. Cardiac outcomes were standardized on body surface area. Cape Verdean, Surinamese-Hindustani, and Turkish children had smaller right ventricular end-diastolic volume and left ventricular end-diastolic volume relative to their body size than Dutch children (*p* < 0.05). These results were not fully explained by fetal and childhood factors. Right ventricular ejection fraction and left ventricular ejection fraction did not differ between ethnicities after adjustment for fetal and childhood factors.

*Conclusion*: Right ventricular end-diastolic volume and left ventricular end-diastolic volume differ between ethnic subgroups in childhood, without affecting ejection fraction. Follow-up studies are needed to investigate whether these differences lead to ethnic differences in cardiac disease in adulthood.

**Table:** 

**What is Known:** • *Ethnic differences in cardiovascular risk factors and disease are well-known and may originate in early-life.* • *The prevalence of cardiovascular disease differs between ethnic groups*. **What is New:** • *We examined ethnic differences in left and right cardiac structure and function in children using cMRI.* • *Right and left cardiac dimensions differ between ethnic groups in childhood and are only partly explained by fetal and childhood factors.*

**What is Known:**

• *Ethnic differences in cardiovascular risk factors and disease are well-known and may originate in early-life.*

• *The prevalence of cardiovascular disease differs between ethnic groups*.

**What is New:**

• *We examined ethnic differences in left and right cardiac structure and function in children using cMRI.*

• *Right and left cardiac dimensions differ between ethnic groups in childhood and are only partly explained by fetal and childhood factors.*

## Introduction

The prevalence of cardiovascular disease differs between ethnic groups [1–4]. Studies in the USA suggest that adults from Hispanic and African American background have higher risks of cardiovascular disease than adults from European background [1, 2]. Similarly, in the UK, cardiovascular disease risk is higher among adults from West-African and South-Asian background than among Caucasians [3, 5]. In the Netherlands, lower cardiovascular mortality was reported among adults from Morocco, but higher mortality was observed among the Surinamese than among native Dutch adults [5]. These possible differences are increasingly relevant with the number of migrants in Western Europe [6, 7].

Differences in common risk factors for cardiovascular disease such as body mass index (BMI), blood pressure, and lipid and insulin concentrations can already be observed in childhood and track to adulthood [8–11]. We previously reported ethnic differences in common cardiovascular risk factors in childhood in the Netherlands, which were mainly explained by early life factors [10]. Although cardiac structures are independent risk factors for mortality in adults, and tend to track from childhood to adulthood, ethnic differences in cardiac structure and function have been studied less extensively [12, 13].

Greater left ventricular end-diastolic volume (LVEDV), left ventricular mass (LVM), and lower LV ejection fraction (LVEF) have been observed in Americans from African background than European background [14]. In the UK, adults from Indian Asian background had smaller LVEDV and LVM than those from European background [15]. Studies in children do not show consistent results [16, 17]. In childhood, lean body mass explains most of the variation in LVM and might differ between ethnicities [10, 18]. Differences in lean mass and other early life factors may influence the ethnic differences in cardiac structure. Most previous studies used echocardiography to evaluate cardiac structures. However, cardiac magnetic resonance imaging (cMRI) has been found to be more accurate and reproducible than echocardiography, and enables studies on volumes of the right side of the heart, another independent risk factor for cardiovascular mortality [19–21].

We examined ethnic differences in left and right cardiac structure and function in children using cMRI in a multi-ethnic population-based study among 2317 children in Rotterdam, the Netherlands, and examined whether any difference was explained by differences in fetal and childhood factors.

## Methods

### Study design

**Fig. 1:**
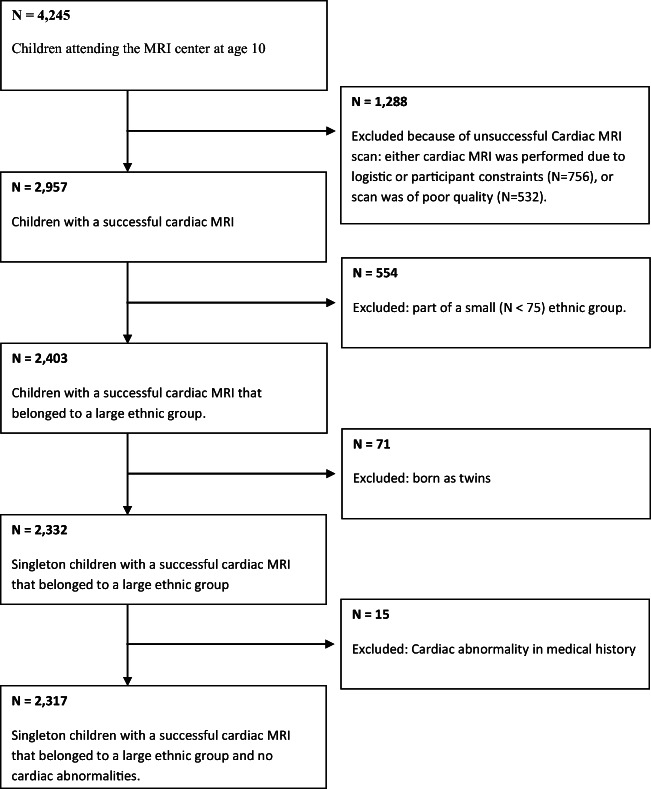
Flow chart of participants included in the analysis

This study was embedded in the Generation R Study, a multi-ethnic population-based prospective cohort study from fetal life until young adulthood in Rotterdam, the Netherlands; we described a general power calculation in the study design paper. For the current explorative analyses, we used a minimum of 50 children per subgroup based on the data availability [22]. The study has been approved by the Medical Ethics Committee of the Erasmus University Medical Center, Rotterdam. Written informed consent was obtained from all parents of participants. At the age of 10, children underwent full-body MRI, including cMRI; because of later start of the MRI studies, not all children within the follow-up period attended the MRI center. A total of 4245 MRI scans were performed during the visit to our research center. cMRI scans were successful for 2957 children. Missing scans were mainly due to poor quality or failure of equipment. We excluded children who were part of a small ethnic group (if *n* < 75) (*n* = 554); because they were twins (*n* = 71); or because they were diagnosed with various cardiac abnormalities (*n* = 15). In total 2317 MRI scans were used in the analysis (Fig. 1). Non-response analyses showed that non-responders were more often boys, of non-Dutch ethnicity, and from lower income families (Supplemental Material Table S1**)**.

### Ethnic background

Ethnic background of the child was defined by the country of birth of the parents, as previously described [10, 22, 23]. We defined the following non-Dutch groups: Cape Verdean (*n* = 85), Dutch Antillean (*n* = 79), Moroccan (*n* = 144), Surinamese (*n* = 173), and Turkish (*n* = 152). Because the Surinamese population consists of persons who originate from Africa (Creoles) and India (Hindustani), we further classified this group into Surinamese-Creole (*n* = 93) or Surinamese-Hindustani (*n* = 80) [24].

### Cardiac magnetic resonance imaging outcomes

We performed cMRI using a wide-bore 3 Tesla scanner (Discovery MR 750, General Electric, Milwaukee, MI, USA), as described in more detail previously [25]. Briefly, we acquired localizer images, followed by ECG gated breath-held scans lasting less than 10 s per breath-hold. A short-axis steady-state free precession (SSFP) cine stack was then obtained with basal slice alignment and covering the ventricles and part of the atria with contiguous 8-mm-thick slices over several end expiration breath-holds. We used a cardiac scanning time of 12 min because of the population-based nature of this study. The scans were stored on a digital archive for post-processing. Off-line image analyses for right and left ventricular measures on the short-axis cine stack were performed by a commercial party and analyzed under supervision of an experienced radiologist (Precision Image Analysis, Kirkland, WA, USA), using Medis QMASS software (Medis, Leiden, the Netherlands). Cardiac measurements included right ventricular end-diastolic volume (RVEDV), right ventricular ejection fraction (RVEF), LVEDV, LVEF, and LVM. We calculated left ventricular mass to volume ratio (LMVR) as LVM/LVEDV.

### Covariates

Information on maternal education, pre-pregnancy body mass index, gestational age, and birth weight was collected by questionnaires and medical charts. Childhood lean body mass percentage was obtained by dual-energy X-ray absorptiometry (DXA) scan (iDXA, General Electric, formerly Lunar Corp., Madison, WI). Percent lean body mass was calculated as lean mass (kg)/body weight (kg). Child height and weight were used to calculate body surface area (BSA), using Haycock’s formula [26]. Child systolic pressure and diastolic blood pressure were measured on the right brachial artery, using the validated automatic sphygmomanometer Accutorr Plus (Datascope Corporation, Fairfield, NJ). These measurements preceded the cMRI visit by a median of 1.1 months (95% range 0–24.8 months).

### Statistical analysis

We constructed BSA adjusted standard deviation scores for the following cardiac outcomes: RVEDV, RVEF, LVEDV, LVEF, and LVM, using the GAMLSS in R version 3.6.1 [27,28]. Since LMVR is not usually normalized on BSA, we created standard deviation scores (SDS) not based on BSA. We used linear regression models to assess the associations of ethnic background with RVEDV, RVEF, LVEDV, LVEF, LVM, and LMVR. The basic models were adjusted for child’s age at the visit, sex, time difference between measurement of BSA and cMRI. If the basic model showed a significant difference, we additionally adjusted for a fetal model which included maternal pre-pregnancy BMI, education level, and gestational age adjusted birth weight, and a childhood model which included all previously mentioned covariates and childhood lean body mass percentage. We started with a wider range of possible covariates based on previous literature. We investigated if those covariates had an effect of > 10% in one or more ethnic groups. In the fetal and childhood, all covariates had a > 10% effect on one of the ethnic groups. Because all variables were highly correlated, adjusting for multiple testing would be too stringent; we did not adjust for multiple testing. However we depicted *p* values as 0.05 and 0.01 level, which enables readers to interpret the significance level. To reduce potential biases related to missing data in covariates, we performed multiple imputation using the MICE packages in R (*n* = 10) [29]. All the analyses were done in R 3.6.1 [27].

## Results

### Subject characteristics

Table 1 shows the subject characteristics. Birth weight was lower in all ethnic groups than in Dutch children, except birthweight of Moroccan children. All ethnic groups, except Moroccan and Surinamese-Hindustani, had higher childhood BSA. The lean mass percentage was lower in all ethnic minorities than in Dutch children, except for the Surinamese-Creole. Table 2 presents the observed cardiac outcomes for the total group and categorized per ethnic group, without taking body size into account. Moroccan children had smaller absolute LVEDV than Dutch children. Surinamese-Hindustani children had smaller RVEDV, LVEDV, and LVM, whereas Surinamese-Creole children had smaller LVEF than Dutch children. Turkish children had smaller RVEDV and LVEDV compared to Dutch children

**Table 1:** Characteristics of the study population ( n = 2317)

	Total *N* = 2317	Dutch *N* = 1706	Cape Verdean *N* = 80	Dutch Antilles *N* = 73	Moroccan *N* = 135	Surinamese-Creole *N* = 93	Surinamese-Hindustani *N* = 79	Turkish *N* = 151
Maternal characteristics								
Pre-pregnancy BMI, median (95% range), kg/m^2^	22.7 (18–35.1)	22.3 (18.1–33.7)	22.3 (19.3–32.3)	23.9 (17.4–44.8)**	26 (17.91–37.18)**	24.2 (17.4–35.2)**	22.9 (17.3–32)	23.5 (18–35.9)**
High education, *n* (%)	1205 (52)	1075 (63)	10 (13)**	13 (18)**	19 (14)**	21 (22)**	20 (25)**	30 (20)**
Birth/infant characteristics								
Male sex, *n* (%)	1112 (48)	819 (48)	45 (56)	31 (42)	63 (47)	49 (53)	38 (48)	74 (49)
Gestational age, median (95% range), kg/m^2^	40.1 (35.9–42.3)	40.3 (36–42.3)	40.1 (36.6–42.0)	40 (33.6–42.0)*	40.4 (36.14–42.57)	39.6 (33–42.1)**	39.9 (35.4–41.8)**	40.2 (34.6–42.1)
Birth weight, mean (SD), g	3454 (563)	3505 (559)	3332 (492)**	3204 (475)**	3500 (549)	3196 (625)**	3078 (498)**	3369 (520)**
Childhood characteristics at 10 years								
Age, median (95% range), year	9.9 (9.4–11.8)	9.9 (9.4–11.8)	10.0 (9.5–11.8)	9.9 (9.5–12)	10.0 (9.63–11.62)	10.0 (9.6–11.9)	10.0 (9.5–11.8)	10.0 (9.6–11.9)
Body surface area, mean (SD), m^2^	1.17 (0.14)	1.16 (0.12)	1.20 (0.16)*	1.24 (0.18)**	1.18 (0.15)	1.22 (0.16)**	1.14 (0.14)	1.22 (0.16)**
Percentage lean body mass, (95% range), %	0.73 (0.6–0.8)	0.74 (0.57–0.85)	0.68 (0.49–0.84)**	0.71 (0.54–0.84)**	0.69 (0.51–0.82)**	0.72 (0.55–0.86)	0.69 (0.53–0.82)**	0.66 (0.53–0.8)**

*BMI*, body mass index; *SD*, standard deviation. Values are means (standard deviation), medians (95% range), or percentages. The values represent the values after multiple imputation (*n* = 10). Differences were estimated using unpaired *t* tests, Mann-Whitney *u* tests, and chi-square tests; Dutch ethnicity was used as reference group. *p value < 0.05; **p value < 0.01

**Table 2:** Cardiac magnetic resonance imaging measurements per ethnic group (n = 2317)

	Total *N* = 2317	Dutch *N* = 1706	Cape Verdean *N* = 80	Dutch Antilles *N* = 73	Moroccan *N* = 135	Surinamese-Creole *N* = 93	Surinamese-Hindustani *N* = 79	Turkish *N* = 151
Right ventricle								
End-diastolic volume, mean (SD), (mL)	100.9 (30.2)	101.4 (26.8)	97.3 (20.8)	104 (21.9)	98.5 (19.5)	101.8 (23.3)	91.5 (20.7)**	96.7 (19.1)**
Ejection fraction, mean (SD), (%)	58.6 (17.9)	58.7 (20.7)	57.7 (5.1)	58.8 (4.8)	58.8 (5)	57.3 (5.6)	57.8 (4.7)	58.6 (4.9)
Left ventricle								
End-diastolic volume, mean (SD), (mL)	101.3 (29)	101.5 (25.4)	98.1 (17.8)	106.5 (21.7)	100.3 (17.9)*	102.1 (20.1)	91.9 (19.3)**	98.2 (18.6)*
Ejection fraction, mean (SD), (%)	58.8 (17.8)	59 (20.6)	57.7 (4.9)	59 (4.6)	58.7 (4.5)	57.3 (4.8)*	58.6 (4.2)	58.9 (4.4)
Mass, mean (SD), g	49.4 (20.2)	48.9 (10)	49.2 (11.2)	51 (10.9)	48.2 (9.1)	52.5 (12.2)	45.6 (10.1)**	49.6 (11.5)
Mass to volume ratio, mean (SD), (g/mL)	0.5 (0.3)	0.5 (0.1)	0.5 (0.1)	0.5 (0.1)	0.5 (0.1)	0.5 (0.1)	0.5 (0.1)	0.5 (0.1)

Values are means (standard deviation). *p* values were estimated using unpaired *t* tests; Dutch ethnicity was used as reference group

*p value < 0.05; **p value < 0.01

### Ethnic background and cardiac outcomes measured by cMRI

Table 3 shows that as compared to Dutch children, relative to their current body size, Cape Verdian children had smaller RVEDV (difference − 0.35 SDS (95% confidence interval (CI) − 0.56, − 0.15)) and LVEDV (difference − 0.33 SDS (95% CI − 0.53, − 0.13)). Surinamese-Hindustani children had smaller RVEDV (difference − 0.51 SDS (95% CI − 0.71, − 0.)) and LVEDV (difference − 0.57 SDS (95% CI − 0.77, − 0.37)). Turkish children had smaller RVEDV (difference − 0.44 SDS (95% CI − 0.59, − 0.29)) and LVEDV (difference − 0.38 SDS (95% CI − 0.53, − 0.23). After adjusting for fetal and childhood factors, the effect estimates attenuated slightly for RVEDV and LVEDV but remained significant in Cape Verdean, Surinamese-Hindustani, and Turkish children. No ethnic differences in RVEF or LVEF were observed.

**Table 3:** Associations of ethnic background with cardiac volume measurements (*n* = 2317)

	Cape Verdean *N* = 80	Dutch Antilles *N* = 73	Moroccan *N* = 135	Surinamese-Creole *N* = 93	Surinamese-Hindustani *N* = 79	Turkish *N* = 151
Right ventricle						
End-diastolic volume						
Basic model^1^	− 0.35 (− 0.56, − 0.15)**	− 0.06 (− 0.28, 0.15)	− 0.16 (− 0.32, 0.01)	− 0.19 (− 0.39, 0.01)	− 0.51 (− 0.71, − 0.3)**	− 0.44 (− 0.59, − 0.29)**
Fetal model^2^	− 0.40 (− 0.65, − 0.15)*	N.A.	N.A.	N.A.	− 0.37 (− 0.6, − 0.13)*	− 0.30 (− 0.48, − 0.13)**
Childhood model^3^	− 0.29 (− 0.54, − 0.04)*	N.A.	N.A.	N.A.	− 0.29 (− 0.52, − 0.06)*	− 0.2 (− 0.38, − 0.03)*
Ejection fraction						
Basic model^1^	− 0.07 (− 0.31, 0.17)	0.19 (− 0.06, 0.44)	0.04 (− 0.15, 0.23)	− 0.09 (− 0.32, 0.13)	− 0.11 (− 0.35, 0.12)	0.09 (− 0.09, 0.26)
Fetal model^2^	N.A.	N.A.	N.A.	N.A.	N.A.	N.A.
Childhood model^3^	N.A.	N.A.	N.A.	N.A.	N.A.	N.A.
Left ventricle						
End-diastolic volume						
Basic model^1^	− 0.33 (− 0.53, − 0.13)**	0.06 (− 0.16, 0.27)	− 0.07 (− 0.23, 0.09)	− 0.21 (− 0.4, − 0.02)*	− 0.57 (− 0.77, − 0.37)**	− 0.38 (− 0.53, − 0.23)**
Fetal model^2^	− 0.36 (− 0.61, − 0.11)*	N.A.	N.A.	− 0.08 (− 0.29, 0.14)	− 0.46 (− 0.69, − 0.23)**	− 0.27 (− 0.45, − 0.10)**
Childhood model^3^	− 0.25 (− 0.5, − 0.01)*	N.A.	N.A.	N.A.	− 0.38 (− 0.61, − 0.15)**	− 0.18 (− 0.35, − 0.01)*
Ejection fraction						
Basic model^1^	− 0.18 (− 0.43, 0.06)	0.17 (− 0.09, 0.42)	0.01 (− 0.18, 0.20)	− 0.16 (− 0.39, 0.07)	0.00 (− 0.24, 0.23)	0.08 (− 0.10, 0.25)
Fetal model^2^	N.A.	N.A.	N.A.	N.A.	N.A.	N.A.
Childhood model^3^	N.A.	N.A.	N.A.	N.A.	N.A.	N.A.

*N.A*., not applicable

Values are linear regression coefficients (95% confidence interval). They represent differences in childhood cardiac structure and function per ethnic group compared to Dutch children (*N* = 1706). Cardiac measures (except mass to volume ratio) were standardized on BSA

All comparisons were made with children from Dutch background as a reference group

^1^Basic models are adjusted for age at MRI scan, sex, time difference, and analyst of the scan. Further adjustments were only performed if the first model was significant

^2^Fetal models are additionally adjusted for: education mother, pre-pregnancy BMI of the mother, and birth weight corrected for gestational age

^3^Childhood models are adjusted for all previously mentioned factors and for percentage of lean body mass

*p value < 0.05; **p value < 0.01

The associations of ethnic background with LVM and LMVR are given in Table 4. Surinamese-Hindustani children had lower LVM (difference − 0.27SDS (95% CI − 0.48, − 0.07)) and higher LMVR (difference 0.78SDS (95% CI 0.50, 1.06)). These differences attenuated fully after adjusting for fetal factors.

**Table 4:** Associations of ethnicity with left ventricle mass measures (n = 2317)

	Cape Verdean *N* = 80	Dutch Antilles *N* = 73	Moroccan *N* = 135	Surinamese-Creole *N* = 93	Surinamese-Hindustani *N* = 79	Turkish *N* = 151
Mass						
Basic model^1^	− 0.17 (− 0.38, 0.04)	− 0.08 (− 0.3, 0.14)	− 0.07 (− 0.24, 0.09)	0.06 (− 0.14, 0.26)	− 0.27 (− 0.48, − 0.07)*	− 0.12 (− 0.27, 0.04)
Fetal model^2^	N.A.	N.A.	N.A.	N.A.	− 0.21 (− 0.45, 0.03)	N.A.
Childhood model^3^	N.A.	N.A.	N.A.	N.A.	N.A.	N.A.
Mass to volume ratio						
Basic model^1^	0.05 (− 0.23, 0.34)	− 0.03 (− 0.32, 0.27)	0.00 (− 0.23, 0.22)	0.08 (− 0.19, 0.35)	0.78 (0.50, 1.06)**	0.08 (− 0.13, 0.28)
Fetal model^2^	N.A.	N.A.	N.A.	N.A.	0.03 (− 0.05, 0.11)	N.A.
Childhood model^3^	N.A.	N.A.	N.A.	N.A.	N.A.	N.A.

*N.A*., not applicable

Values are linear regression coefficients (95% confidence interval). They represent differences in childhood cardiac structure and function per ethnic group compared to Dutch children (*N* = 1706). Cardiac measures (except mass to volume ratio) were standardized on BSA

All comparisons were made with children from Dutch background as a reference group

^1^Basic models are adjusted for age at MRI scan, sex, and the time difference between measuring of anthropometrics and cMRI. Further adjustments were only performed if the first model was significant

^2^Fetal models are additionally adjusted for: education mother, pre-pregnancy BMI of the mother, and birth weight corrected for gestational age

^3^Childhood models are adjusted for all previously mentioned factors and for percentage of lean body mass

*p value < 0.05; **p value < 0.01

## Discussion

In this multi-ethnic population-based prospective cohort study among 10-year-old children born in the Netherlands, children from Cape Verdean, Surinamese-Hindustani, and Turkish background had smaller RVEDV and LVEDV, relative to their current BSA than Dutch children. These differences were only partly explained by fetal and childhood factors. We did not find consistent associations of ethnicity with RVEF, LVEF, LVM, or LMVR after adjusting for fetal or childhood factors.

Several studies have shown a difference in cardiovascular disease between ethnic groups [1–5]. In the Netherlands, higher cardiovascular mortality has been observed in adults of Surinamese background [5]. Adults from Moroccans background generally show lower risk of cardiovascular mortality [5]. Although self-reported cardiovascular disease was higher in adults from Turkish background, studies on cardiovascular outcomes have reported conflicting results [5,30].

In our current study, we observed lower RVEDV and LVEDV in children from Cape Verdean, Surinamese-Hindustani, and Turkish background, as compared to Dutch children. The differences in RVEDV and LVEDV in children from Cape Verdean, Surinamese-Hindustani, and Turkish background attenuated slightly after adjustment for fetal and childhood factors. In childhood, cardiac size is mainly determined by lean body mass [18]. Although we standardized the cardiac outcomes on BSA and adjusted for lean body mass percentage, other unaccounted factors of body size and composition might explain part of the differences in cardiac structure that we observed. Also, it is known that well-trained athletes have a larger LVEDV [31]. In the current study, we do not have detailed information on cardiovascular fitness and overall physical activity available, and we were not able to examine if differences in fitness could explain the associations of ethnicity with cardiac structure.

In adults a larger heart and higher LVM as well as RVEDV and LVEDV, are generally associated with higher risk of cardiovascular disease [12, 32]. A higher LMVR is associated with adverse cardiac outcomes, independent from LVM [33]. We observed a lower LVM and a higher LMVR in children from Surinamese-Hindustani background compared to Dutch children, but these associations attenuated fully after adjustment for fetal factors.

Our findings suggest that ethnicity is associated with cardiac structure at school-age, and this association is only partly explained by fetal and childhood factors. The differences we observed were small, and within normal ranges. Further studies are needed to explore whether these differences relate to adult cardiovascular disease. Multiple studies have established Z-scores for cardiac MRI. However, many of these studies are based on a relatively small study population. It would be interesting to create normal values in a large study population across all age-groups taking account for differences between ethnic subgroups.

To our knowledge, this is the first study focused on the associations of children’s ethnicity with cardiac structure and function using cMRI in a large and diverse multi-ethnic prospective cohort. The major advantages of cMRI instead of echocardiography are more accurate measurements and information about the right cardiac structures [19]. Some limitations need to be considered. Of the original cohort, 60% of the children visited the research center at the age of 10 years. Of these children, 72% also attended the MRI center. Lower attendance is due to later start of the MRI study. cMRI was not performed in 15% of children that underwent body MRI. Reasons for not performing cMRI were often logistical, such as malfunctioning of equipment or time constraints. Also, we were not able to analyze 16% of the cMRI scans due to poor quality of the scans. This was partly due to using an 3.0T MRI scan, which is less ideal for imaging of the body [20, 19]. Height and weight used to calculate BSA were measured during an earlier visit than the MRI scan. Around 65% of children visited the MRI within 2 months after the BSA visit, with a median time difference of 1.28 months (95% range (0, 24.2)). Even though we adjusted for the time difference, measurement error can cause attenuation of the observed associations. Finally, it is possible that the observed differences are explained by residual confounding. This could be due to specific factors such as physical activity, blood pressure and arterial hypertension, clustering of cardiovascular risk factors diet or possible comorbidities such as diabetes mellitus or chronic kidney disease (either diagnosed or subclinical), hyperlipidemia, or parental smoking exposure or other parental and childhood lifestyle characteristics [34, 35]. We previously reported ethnic differences in well-known cardiovascular risk factors [10]. Adjustment for these factors did not explain all ethnic differences in cardiovascular risk factors.

## Conclusions

Right and left cardiac dimensions differ between ethnic groups in childhood. These differences are only partly explained by fetal and childhood factors. Further research is necessary to examine how ethnic differences in cardiac structure progress during and after adolescence, and if this contributes to later cardiovascular disease.

## Data Availability

Data is available upon request from the principal investigator (Prof. Dr. Vincent Jaddoe)

## Abbreviations

BMI: Body mass index
BSA: Body surface area
cMRI: Cardiac magnetic resonance imaging
LVEF: Left ventricular ejection fraction
LVEDV: Left ventricular end-diastolic volume
LVM: Left ventricular mass
LMVR: Left ventricular mass to volume ratio
RVEF: Right ventricular ejection fraction
RVEDV: Right ventricular end-diastolic volume
